# Blocking Activin Receptor Ligands Is Not Sufficient to Rescue Cancer-Associated Gut Microbiota—A Role for Gut Microbial Flagellin in Colorectal Cancer and Cachexia?

**DOI:** 10.3390/cancers11111799

**Published:** 2019-11-15

**Authors:** Satu Pekkala, Anniina Keskitalo, Emilia Kettunen, Sanna Lensu, Noora Nykänen, Teijo Kuopio, Olli Ritvos, Jaakko Hentilä, Tuuli A. Nissinen, Juha J. Hulmi

**Affiliations:** 1 Faculty of Sport and Health Sciences, University of Jyväskylä, 40620 Jyväskylä, Finland; emilia.l.kettunen@gmail.com (E.K.); sanna.t.k.lensu@jyu.fi (S.L.); jaakko.j.hentila@jyu.fi (J.H.); tuuli.a.m.nissinen@jyu.fi (T.A.N.); juha.j.t.hulmi@jyu.fi (J.J.H.); 2 Institute of Biomedicine, Faculty of Medicine, University of Turku, 20500 Turku, Finland; anniina.keskitalo@utu.fi; 3 Department of Clinical Microbiology, Turku University Hospital, 20500 Turku, Finland; 4 Department of Pathology, Central Finland Health Care District, Keskussairaalantie 19, 40620 Jyväskylä, Finland; noora.nykanen@ksshp.fi (N.N.); teijo.kuopio@ksshp.fi (T.K.); 5 Department of Biological and Environmental Science, University of Jyväskylä, 40620 Jyväskylä, Finland; 6 Department of Physiology, Faculty of Medicine, University of Helsinki, 00100 Helsinki, Finland; olli.ritvos@gmail.com

**Keywords:** inflammation, activin, myostatin, microbiome, IL6, CCL2, MCP-1

## Abstract

Colorectal cancer (CRC) and cachexia are associated with the gut microbiota and microbial surface molecules. We characterized the CRC-associated microbiota and investigated whether cachexia affects the microbiota composition. Further, we examined the possible relationship between the microbial surface molecule flagellin and CRC. CRC cells (C26) were inoculated into mice. Activin receptor (ACVR) ligands were blocked, either before tumor formation or before and after, to increase muscle mass and prevent muscle loss. The effects of flagellin on C26-cells were studied in vitro. The occurrence of similar phenomena were studied in murine and human tumors. Cancer modulated the gut microbiota without consistent effects of blocking the ACVR ligands. However, continued treatment for muscle loss modified the association between microbiota and weight loss. Several abundant microbial taxa in cancer were flagellated. Exposure of C26-cells to flagellin increased *IL6* and *CCL2/MCP-1* mRNA and IL6 excretion. Murine C26 tumors expressed more *IL6* and *CCL2/MCP-1* mRNA than C26-cells, and human CRC tumors expressed more CCL2/MCP-1 than healthy colon sites. Additionally, flagellin decreased caspase-1 activity and the production of reactive oxygen species, and increased cytotoxicity in C26-cells. Conditioned media from flagellin-treated C26-cells deteriorated C2C12-myotubes and decreased their number. In conclusion, cancer increased flagellated microbes that may promote CRC survival and cachexia by inducing inflammatory proteins such as MCP-1. Cancer-associated gut microbiota could not be rescued by blocking ACVR ligands.

## 1. Introduction

An increasing number of studies are reporting that an altered gut microbiota is associated with various types of cancer, especially colorectal cancer (CRC) [1]. The gut microbiota refers to the ~100 trillion microbial cells inhabiting our gastrointestinal tract, outnumbering the human cells by ~1.3-fold [2], with whom humans have been living for millions of years in a symbiotic, mutualistic relationship. The gut microbiota, for instance, strengthens our immune system and breaks down macronutrients from ingested food into by-products that affect the host’s metabolism and inflammatory status [3]. Certain gut microbes have been described to promote cancer through various mechanisms, including the production of toxins, harmful metabolites, inflammation, and oxidative stress [4]. An accumulation of knowledge on the microbial signatures in cancer also raised the hope that the gut microbiota could be targeted to treat cancer-associated cachexia. Cachexia is a complex multi-organ syndrome characterized by involuntary weight loss, muscle atrophy, anorexia, and inflammation [5]. Cachexia-associated gut microbiota, however, has only been studied in preclinical animal models [6]. A recent study shed light on the possible role of the gut microbiota in human cachexia by showing that cachectic patients had higher serum levels of bacterial lipopolysaccharide binding protein. Therefore, the study provided an indication of increased gut permeability in cachectic patients compared with non-cachectic cancer patients [7]. The intestinal barrier prevents the entry of pathogenic micro-organisms and toxic substances into the circulation, and thus an increased gut permeability often leads to chronic, systemic, low-grade inflammation that may be caused by the leakage of entire bacteria or their surface molecules, such as lipopolysaccharides [8].

CRC is among the most commonly diagnosed cancers, one of the leading causes of cancer death worldwide [9], and a disease in which the prevalence of cachexia is ~30–50%. The prognosis of CRC depends largely on the tumor location; right-sided tumors generally have worse prognoses [10]. In right-sided CRC, elevated tumor lymphocytic infiltration and an inflammatory microenvironment [11], as well as dense gut microbial biofilms (bacterial aggregates), are present [12]. Biofilms increase the production of interleukin 6 (IL6), suggesting that the gut microbiota may also induce the secretion of IL6 by the tumor. IL6 is associated with poor prognosis for both CRC overall survival and disease-free survival [13]. In addition to IL6, monocyte chemoattractant protein 1 (MCP-1/CCL2) has been linked to intestinal tumorigenesis [14] and to cachexia in human cancer patients [15]. Altogether, this inflammatory microenvironment in CRC can cause a breakdown of the colonic epithelial barrier and leakage of inflammation-inducing gut microbial compounds into the circulation.

One of these microbial compounds is the bacterial flagellum, which is primarily a locomotive organelle, but also an important mechanosensor that initiates the formation of biofilms [16]. Therefore, it might link microbial biofilms and the gut microbiota to CRC. Supporting the role of flagellum in CRC, a study on European population reported that exposure to bacterial flagellin, a structural protein of flagellum, was positively associated with CRC risk [17]. However, there are also contradictory findings on the role of flagellin in cancer. These studies have shown either a potent tumor suppressing effect [18], an induction of tumor growth [19], or an induction of chemoresistance in multiple myeloma cells [20].

Flagellin, like other pathogen-associated molecular patterns, is recognized by pattern recognition receptors to initiate innate immune responses. Extracellular flagellin binds to the Toll-like receptor 5 (TLR5) on the cell surface, whereas flagellin that reaches the cytosol is identified by the NOD-like receptor (NLR) family CARD domain-containing protein 4 (NLRC4), which further recruits the NLR family pyrin domain-containing 3 (NLRP3) [21]. The role of TLRs in cancer is not without controversy. On one hand, potent TLR agonists are used to fight cancer as immune adjuvants, whereas on the other hand, TLRs can induce inflammation, programmed necrosis, and autophagy [22]. These events in concert can promote tumor survival and the formation of metastases [23]. In addition, TLR7 has been shown to promote tumor progression through the recruitment of myeloid-derived suppressor cells (MDSC) [24].

In this study, we aimed to characterize cancer and cachexia-associated gut microbiota composition in a preclinical cancer model, in which C26 colon cancer cells [25] were inoculated subcutaneously into syngeneic mice, inducing cancer cachexia [26]. Moreover, the possible importance of cachexia or cancer-induced muscle loss per se on the gut microbiota was investigated experimentally by administering a systemic blocker of activin receptor 2B ligand (sACVR) to the C26 tumor-bearing mice that is able to alleviate muscle loss and improve survival in C26 cancer [26,27]. After observing that several flagellated microbes were abundant in the tumor-bearing mice, we further characterized the direct in vitro effects of the gut microbiota-derived flagellin on mouse C26 colon cancer cells and MDSC-mimicking human acute monocytic leukemia cell line, THP-1. The possible contribution of flagellin to the phenomena associated with cancer cachexia was studied in vitro using C26 cells and murine C2C12 myotubes. Finally, we retrospectively determined whether the phenomena mimicking those induced by flagellin in vitro can be detected in murine and human tumors.

## 2. Results

### 2.1. Experimental C26 Cancer Increased the Diversity of the Gut Microbiota Compared to Healthy Mice without Consistent Effects of Blocking Activin Receptor Ligands

**Figure 1:**
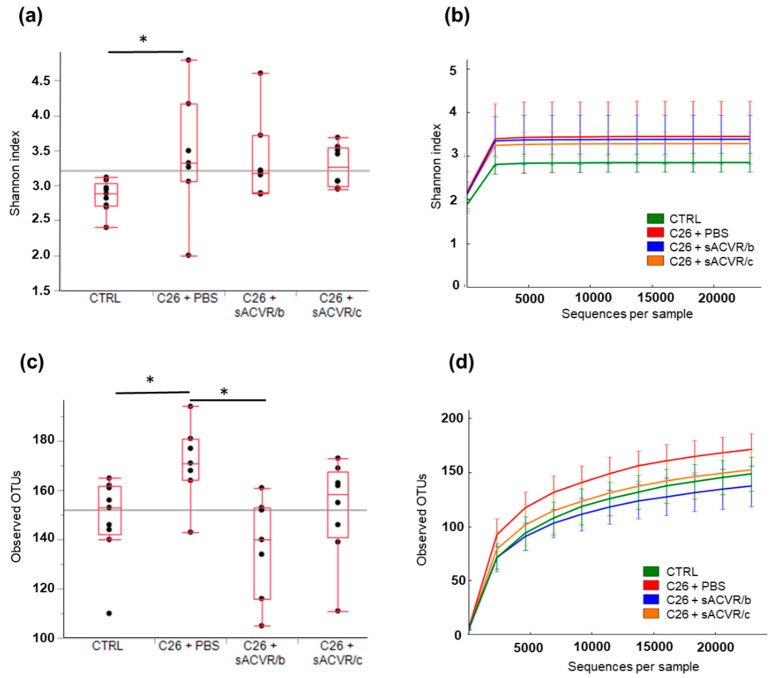
Alpha-diversity of the mice gut microbiota samples. (**a**) Based on average Shannon indices, or (**b**) Shannon indices shown as sequences per sample, the microbiota of the control (CTRL) samples was significantly less diverse than in the other groups. (**c**) The average operational taxonomic unit (out) richness and (**d**) the OTUs shown as sequences per sample of the C26 + PBS group gut microbiota tended to be higher than in other samples. Phosphate buffered saline (PBS), a systemic blocker of activin receptor 2B ligands (sACVR2B), Control (CTRL, *n* = 9), cancer (C26 + PBS, *n* = 7), the group that received sACVR2B until tumor formation (C26 + sACVR/b, *n* = 7), and the group that received sACVR2B until death (C26 + sACVR/c, *n* = 8). * Denotes statistically significant difference between the groups.

The experimental C26 cancer induced cachexia, which was indicated by substantial muscle and fat mass loss, as previously shown by us [26]. By blocking activin receptor ligands using a soluble ligand trap (sACVR), the muscle loss was partly prevented [26]; other cachectic features, such as splenomegaly and anemia, were also blocked [26]. The bacterial diversity of the stool samples, represented as average Shannon index values, was significantly higher in the untreated cancer group (C26 + phosphate buffered saline (PBS) than in the control group (CTRL) (*p* < 0.05). Neither treating the mice with sACVR until tumor formation (C26 + sACVR/b), nor both before and after tumor formation (C26 + sACVR/c) were able to prevent this response (Figure 1a,b). The bacterial richness, i.e., the observed different operational taxonomic units (OTUs) per sequences (Figure 1c,d), was significantly higher in the C26 + PBS group than in the CTRL and C26 + sACVR/b group (*p* = 0.015 and *p* = 0.007, respectively; Figure 1c,d). Because the Shannon index scales the OTU numbers based on the evenness of the communities, these results suggest that the OTU distribution of the CTRL and C26 + PBS was skewed, reducing the Shannon index of these samples, while the OTUs of the mice that had the continued treatment for cachexia (C26 + sACVR/c) were more evenly distributed.

**Figure 2:**
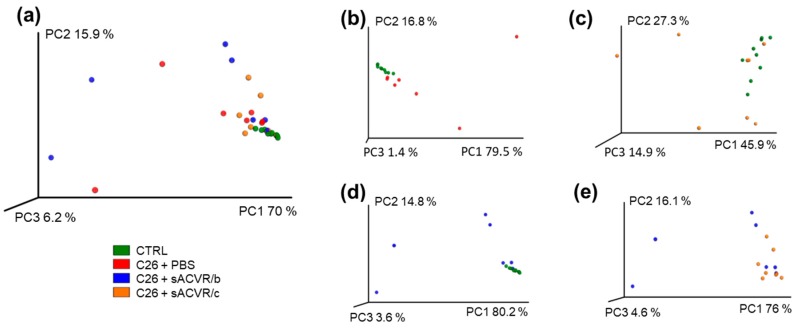
Beta-diversity of the mice microbiota samples. No clear differences were visually observed between the groups in principal component analysis (PCA) plot. (**a**) PCA plot of all groups; (**b**) PCA plot of groups CTRL and C26 + PBS; (**c**) PCA plot of groups CTRL and C26 + sACVR/c; (**d**) PCA plot of groups CTRL and C26 + sACVR/b; (**e**) PCA plot of groups C26 + sACVR/b and C26 + sACVR/c. Control (CTRL, *n* = 9), cancer (C26 + PBS, *n* = 7), the group that received sACVR2B until tumor formation (C26 + sACVR/b, *n* = 7), and the group that received sACVR2B until death (C26 + sACVR/c, *n* = 8).

In the principal coordinate analysis (PCA) plot where the individual samples with similar microbiota composition cluster together, the CTRL samples clustered clearly together, representing a strong homology within the group, while in all other groups, clear inter-individual differences occurred, especially in the C26 + PBS and CTRL and C26 + sACVR/b groups (Figure 2a). However, based on adonis analysis, the sample grouping explained approximately 24% of the overall clustering in the PCA plot (*p* = 0.011). The level of explanation was 27.3% (*p* = 0.001) between the CTRL and C26 + PBS (Figure 2b), 14.8% (*p* = 0.029) between the CTRL and C26 + sACVR/c (Figure 2c), 27.4% (*p* = 0.003), between the CTRL and C26 + sACVR/b (Figure 2d), and 17.7% (*p* = 0.039) between the C26 + sACVR/c and C26 + sACVR/b (Figure 2e). The C26 + PBS did not differ from the sACVR-treated groups, meaning that the treatment did not explain the clustering of the samples in the PCA plot.

### 2.2. C26 Cancer, but Not the Alleviation of Cachexia by sACVR Administration, Modulated the Gut Microbiota Composition

**Figure 3:**
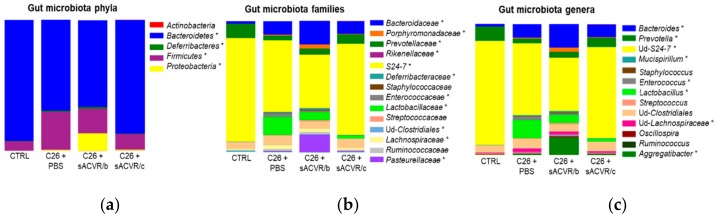
The gut microbiota composition of the mice at (**a**) phylum, (**b**) family, and (**c**) genus level. * Denotes significantly differing taxa between the CTRL and C26 + PBS group (False Discovery Rate (FDR) < 0.05). None of the sACVR administration protocols had significant effects (no difference compared to the C26 + PBS group). The gut microbiota compositions at the phylum level are shown on the left side, the family level in the middle, and the genus level on the right side. Control (CTRL, *n* = 9), cancer (C26 + PBS, *n* = 7), the group that received sACVR2B until tumor formation (C26 + sACVR/b, *n* = 7), and the group that received sACVR2B until death (C26 + sACVR/c, *n* = 8).

The treatment of cachexia by sACVR administration did not significantly affect any microbial taxa (no difference between the sACVR-treated groups vs. C26 + PBS) despite the fact that the gut microbiota composition in the C26 + sACVR/b group seemed to differ from the other groups (Figure 3). In addition, while the C26 + sACVR/c group seemed to resemble controls more than C26 + PBS, the differences between the groups were not significant. This indicates that the muscle atrophy per se did not affect the microbiota composition, while cancer did. The most dominant phylum in all four groups was Bacteroidetes, representing on average 66% to 93% of the sequences. The most dominant family and further an unidentified genus was S24-7, representing on average 80% of the sequences in the CTRL and 55% in C26 + PBS group. Several phylum-, family-, and genus-level differences in the taxa between the CTRL and C26 + PBS were found (Figure 1, Table 1). An interesting notion, though not significantly different from the C26 + PBS, was that the sACVR/b-treated mice had unusually high relative abundances of *Aggregatibacter* (13% of the sequences) and Porphyromonadaceae (3%), which include common oral pathogens. The relative abundance of Porphyromonadaceae was 0.5% in C26 + PBS, 0.4% sACVR/c, and undetectable in the CTRL mice. Of the increased taxa in cancer, for instance *Mucispirillum*, represented by a single species *Mucispirillum schaedleri*, is flagellated [28]. In addition, Proteobacteria [29], *Lachnospiraceae* [30], *Enterococcus* [31], and *Bacteroides* [32] contain several flagellated species, although not all their representatives are motile.

### 2.3. Association of the Gut Microbiota with Cancer Cachexia-Associated Features

**Figure 4:**
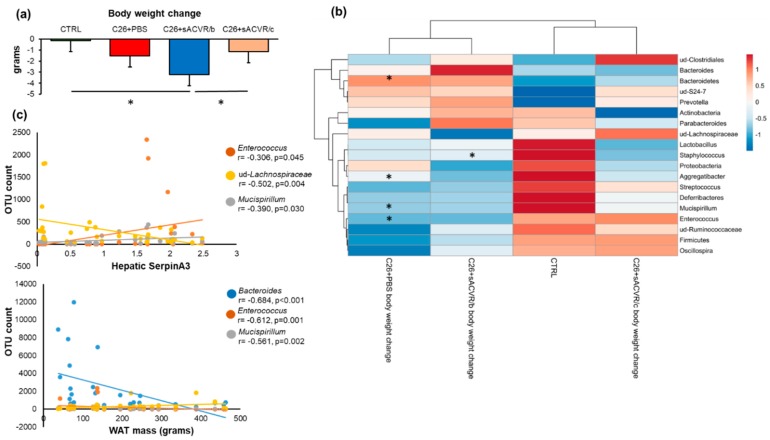
(**a**) The body weight change during the last three days before necropsy. (**b**) Several microbial phyla and genera were associated with the body weight loss, but not significantly in the CTRL and C26 + sACVR/c groups. In the heat map analysis using correlation distance and average linkage, the C26 + PBS and C26 + sACVR/b groups clustered together, indicating that the associations between body weight loss and the gut microbiota in the C26 + PBS and C26 + sACVR/b groups resembled each other and differed from the CTRL and C26 + sACVR/c groups. The FDR-corrected significant associations and differences between the groups in body weight change are marked with asterisks (*). The numeric colored scale bar at upright corner represents the Spearman correlation coefficients. (**c**) The flagellated gut microbial taxa and their association with the cachectic features of hepatic expression of acute-phase response protein SerpinA3 and white adipose tissue (WAT) mass. Control (CTRL, *n* = 9), cancer (C26 + PBS, *n* = 7), the group that received sACVR2B until tumor formation (C26 + sACVR/b, *n* = 7), and the group that received sACVR2B until death (C26 + sACVR/c, *n* = 8).

During the last three days before necropsy, body weight decreased more in the C26 + sACVR/b group in which the treatment was discontinued after the cancer cell inoculation when compared to the healthy CTRL mice (*p* < 0.001) and C26 + sACVR/c group in which the sACVR-treatment was continued after cancer cell inoculation (Figure 4a). Several microbial taxa were associated with body weight loss, but not significantly in the CTRL and C26 + sACVR/c groups (Figure 4b). When the groups were analyzed together in the correlation analysis, all significant associations remained except unidentified Ruminococcacceae (data not shown). Despite no differences being observed in the taxa between the C26 + PBS and sACVR-treated mice, in the heat map analysis using correlation distance and average linkage, the C26 + sACVR/c group clustered together with CTRL, while C26 + PBS and C26 + sACVR/b clustered together (Figure 4b). These results indicate that the associations between body weight loss and the gut microbiota in the C26 + PBS and C26 + sACVR/b groups resembled each other and differed from the CTRL and C26 + sACVR/c groups. We also analyzed whether flagellated members of the gut microbiota genera were associated with other cachectic features reported earlier [26]. No associations between the taxa and the weight of spleen were found (data not shown). Subcutaneous white adipose tissue (WAT) mass was associated with *Bacteroides* (r = −0.608, *p* ≤ 0.001), *Mucispirillum* (r = −0.561, *p* = 0.002), and *Enterococcus* (r = −0.612, *p* = 0.001) (Figure 4c), and the hepatic expression of acute-phase response protein SerpinA3 with *Mucispirillum* (r = −0.390, *p* = 0.030), unidentified (ud) *Lachnospiraceae* (r = −0.502, *p* = 0.004), and *Enterococcus* (r = −0.362, *p* = 0.045) (Figure 4c). In addition, ud-*Lachnospiraceae* associated with the hepatic expression of acute-phase response fibrinogen (r = −0.533, *p* = 0.003). As reported earlier, the cancer-induced hepatic acute-phase response was not prevented by the sACVR treatment, unlike splenomegaly [26].

### 2.4. Gut Microbiota-Derived Flagellin Increased Inflammation in C26 Colon Cancer Cells In Vitro

**Figure 5:**
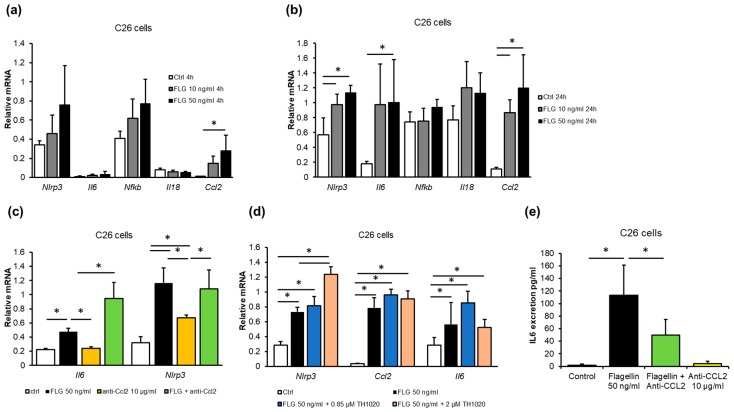
Flagellin exposure of C26 cancer cells (*n* = 4 per treatment) increased inflammation dose-dependently (**a**) after 4 h and (**b**) after 24 h. (**c**) The effects of flagellin on mRNA levels were not affected by blocking CCL2 with neutralizing antibody (anti-CCL2) or (**d**) TLR5 antagonist TH1020 (*n* = 4 per treatment). (**e**) Flagellin exposure increased IL6 excretion from C26 cells that was blocked by CCL2-neutralizing antibody (*n* = 4 per treatment). * Denotes a statistically significant difference between the groups that are connected with the line. Ctrl, control; FLG, flagellin.

A recent epidemiological study suggested that exposure to gut microbiota-derived flagellin was associated with CRC [17]. Interestingly, in our study, several abundant taxa in the experimental cancer included motile, i.e., flagellated, microbes, such as Proteobacteria (Figure 3). We therefore sought to determine whether flagellin exposure affects mouse C26 cells in vitro. Flagellin induced inflammation time- and dose-dependently. After 4 h of exposure, 50 ng/mL of flagellin increased the mRNA of (CCL2/MCP-1) (*p* = 0.016, Figure 5a), and after 24 h of NLRP3 (*p* = 0.003), CCL2/MCP-1 (*p* = 0.004) and IL6 (*p* = 0.038, Figure 5b). After 24 h, 10 ng/mL of flagellin also increased NLRP3 (*p* = 0.020) and CCL2/MCP-1 (*p* = 0.029). Flagellin did not affect the expression of IL18 or nuclear factor kappa B (Nfkb) mRNA (Figure 5a,b).

IL6 is known to be regulated by CCL2 [33], thus, flagellin was used to challenge the cells together with CCL2-neutralizing antibody to find out whether the effects of flagellin were dependent on CCL2. Flagellin increased the mRNA of IL6 (*p* = 0.02) and NLRP3 (*p* = 0.001), independent of CCL2 blocking (Figure 5c). To study whether the effects of flagellin were mediated by TLR5, the receptor was blocked with a specific antagonist, TH1020 [34]. The antagonist had no major effects, indicating that the mRNA expression changes in response to flagellin were not mediated by TLR5 (Figure 5d). The increased IL6 mRNA due to flagellin exposure was accompanied by a ~100-fold increase in IL6 in the C26 cell culture media (*p* = 0.002, Figure 5e). Blocking CCL2 with the neutralizing antibody attenuated the increase in IL6 (*p* = 0.003 compared to flagellin, Figure 5e), indicating that CCL2 was involved in the flagellin-induced excretion of IL6 from the C26 cells.

**Figure 6:**
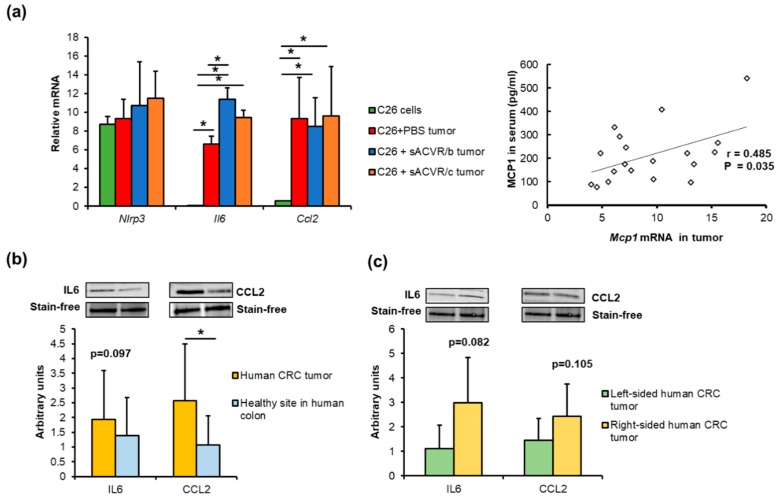
(**a**) Compared to the non-implanted C26 cancer cells, mouse C26 tumors expressed more CCL2 and IL6, independent of the sACVR treatment. The expression of CCL2/MCP-1 in the tumors was associated with the level of MCP-1 in sera. (**b**) Human CRC tumors (*n* = 13) expressed more CCL2 than the healthy colon sites. (**c**) The right-sided tumors (*n* = 6) tended to have higher expression levels of IL6 and CCL2 than the left-sided tumors (*n* = 7). * Denotes a statistically significant difference between the groups that are connected with lines. Control (CTRL, *n* = 9), cancer (C26 + PBS, *n* = 7), the group that received sACVR2B until tumor formation (C26 + sACVR/b, *n* = 7), and the group that received sACVR2B until death (C26 + sACVR/c, *n* = 8).

Unfortunately, in vivo measurement of flagellin levels is challenging, in part because its appearance in blood and organs is sporadic and transient [17,35]. Therefore, from the mice and human tumors, we could only retrospectively determine whether phenomena similar to those induced by flagellin in vitro could be detected. This was done by comparing (i) murine cancer cells with the tumor tissue, (ii) human CRC tumors with the healthy colon tissue, and (iii) human tumors based on their potential aggressiveness and sidedness. Compared to the C26 cells, the mouse C26 tumors expressed more IL6 (*p* < 0.001) and CCL2/MCP-1 (*p* = 0.006), independent of the sACVR treatment (Figure 6a). We previously showed that MCP-1 was also increased in the sera of the C26 cancer mice [26], and here we found that the expression of CCL2/MCP-1 in the tumors was associated with the levels of MCP-1 in sera (R = 0.485, *p* = 0.035, Figure 6b). The sACVR/b-treated mice had higher expression levels of IL6 than the untreated C26 + PBS mice (*p* = 0.003, Figure 6a). In humans, the expression of CCL2 was higher in CRC tumors than in the specimen taken from a healthy site of the colon during surgery (*p* = 0.021, Figure 6b). This trend was similar in IL6 (*p* = 0.097, Figure 6b). The inflammatory microenvironment is suggested to be more “aggressive” in the right-sided CRC tumors than in the left-sided tumors [11]. We therefore also compared the protein expression levels of the right- and left-sided tumors. Due to the heterogeneity and small number of samples per group, the difference did not reach statistical significance, but the right-sided tumors tended to have higher levels of IL6 (*p* = 0.082, Figure 6c) and CCL2 compared to the left-sided ones (*p* = 0.105, Figure 6c). Whether the patient had adenoma or adenocarcinoma did not significantly affect the results.

### 2.5. Flagellin Inhibited Caspase-1 but Not Caspase-8 Activity, Increased Cytotoxicity Independent of Receptor-Interacting Protein (RIP) Kinases, and Decreasedreactive Oxygen Species (ROS) Levels in C26 Cancer Cells In Vitro

**Figure 7:**
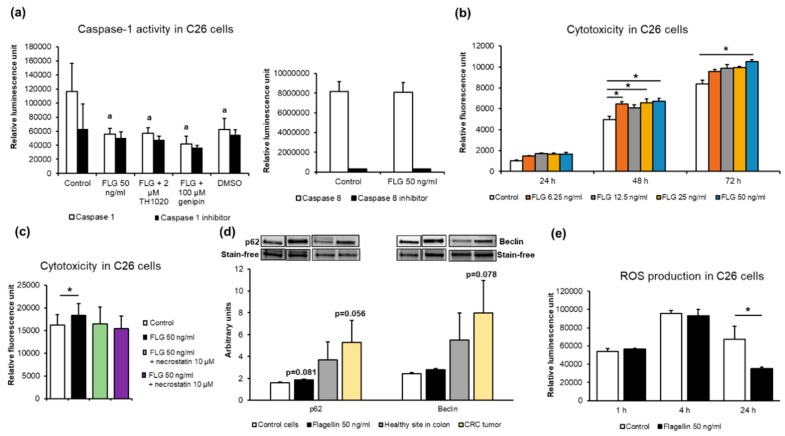
Flagellin (FLG) exposure (**a**) decreased C26 cancer cell apoptosis, i.e., caspase-1 activity (*n* = 4 per treatment), but not caspase-8 activity. (**b**) Flagellin increased cytotoxicity (*n* = 4 per treatment). (**c**) Flagellin-induced cytotoxicity was not affected by the Receptor-interacting protein (RIP) kinase inhibitor necrostatin in C26 cells. (**d**) The expression of the autophagy marker p62 tended to be increased in flagellin-treated C26 cells and CRC tumors compared to controls. (**e**) Flagellin exposure for 24 h decreased ROS levels in C26 colon cancer cells (*n* = 4 per treatment). a = statistically significant difference compared to control and * denotes a significant difference between the groups that are connected with lines. FLG = flagellin; TH1020 = TLR5 antagonist; ROS, reactive oxygen species.

Due to the flagellin-induced IL6, which is known to inhibit apoptosis in many cancer cell types [36], we measured the effects of flagellin on the caspase-1 activity of the C26 cells together with either TLR5 antagonist or genipin, which inhibits NLRP3-mediated inflammasome activation [37]. Compared to the vehicle-treated controls, flagellin decreased the caspase-1 activity (*p* = 0.002, Figure 7a), which was not affected by the addition of TLR5 antagonist or genipin.

Dimethyl sulfoxide (DMSO) as a vehicle for the antagonist and genipin (not for flagellin) decreased caspase-1 activity to the same extent as the caspase 1-specific inhibitor Ac-YVAD-CHO (*p* = 0.03). This was not unusual, because DMSO has been reported to also down-regulate apoptosis in hepatocytes [38]. Flagellin did not affect caspase-8 activity in C26 cells (Figure 7a).

Cancer cells that manage to evade apoptosis tend to undergo necrosis or necroptosis and autophagy that can promote tumor growth and survival [39]. Necrosis is associated with unprogrammed cell death resulting from cellular damage or infiltration by pathogens, while necroptosis is programmed via NOD-like receptors and RIP-kinases [40]. We therefore sought to determine whether the prolonged exposure of C26 cells to different concentrations of flagellin increased cytotoxicity, and whether RIP-kinases were involved. Compared to the controls, 6.25, 25 and 50 ng/mL of flagellin increased cytotoxicity at 24 h and 50 ng/mL at 72 h (*p* < 0.05 for all, Figure 7b). To evaluate whether RIP-kinases were involved in the cytotoxicity, the cells were treated with 50 ng/mL of flagellin together with the RIP-kinase inhibitor necrostatin. The addition of necrostatin did not affect the cytotoxicity (Figure 7d), indicating that the flagellin-induced cell death was likely necrotic and not necroptotic. To assess the expression of autophagy-related proteins, we determined the expression levels of Beclin and p62, which would link the autophagy pathway to the ubiquitin–proteasome system [41]. Unfortunately, microtubule-associated protein light chain 3 (LC3) and Ser757-phosphorylated Unc-51-Like Autophagy Activating Kinase 1 (ULK) could not be reliably detected. Though not statistically significant, flagellin exposure of C26 cells tended to increase p62 (*p* = 0.08, Figure 7c), and human CRC tumors tended to express more p62 and Beclin than the healthy colon sites (*p* = 0.056 and 0.078, respectively, Figure 7c). Finally, flagellin exposure for 24 h decreased the levels of ROS in C26 cells (*p* = 0.021, Figure 7d). Altogether, these results indicate that flagellin inhibited caspase-1 activity and production of ROS, and increased necrosis in C26 cancer cells.

### 2.6. The Conditioned Media of Flagellin-Treated C26 Cells Appeared to Deteriorate Murine C2C12 Myotubes and Decreased Their Number

**Figure 8:**
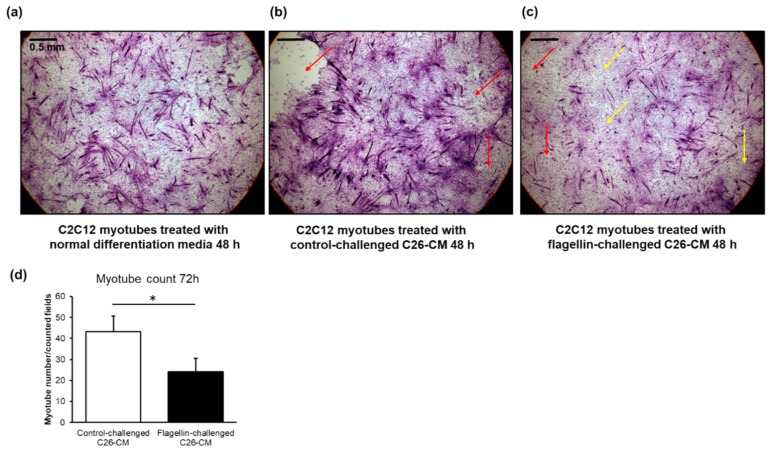
(**a**) Compared to the C2C12 murine myotubes growing in normal differentiation media and (**b**) conditioned media of the C26 cells with control challenge, (**c**) the conditioned media of the flagellin-challenged C26 cells deteriorated and dedifferentiated the multinucleated myotubes into mononucleated myoblasts after 48 h of exposure. The images are hematoxylin & eosin stainings at 4× magnification. The scale bar is 0.5 mm. Red arrows point to the field where myotubes are detached. Yellow arrows point to areas where the myotubes are dedifferentiated. (**d**) Compared to the exposure with the conditioned media of vehicle-treated C26 cells (control), the media of flagellin-treated cells decreased the number of C2C12 myotubes after 72 h of exposure. The scoring of myotubes vs. dedifferentiated myoblasts was based on whether they were multinucleated or mononucleated, respectively. The number of myotubes in each coverslip was counted manually using three to five (depending on the extent of detachment of myotubes) random fields that with 4× magnification, which practically covered the entire coverslip. CM = conditioned media. * Denotes statistically significant difference.

To assess the possible role of flagellin in cancer-associated cachexia in vitro, C26 cells were treated with vehicle or flagellin for 24 h and the conditioned media was collected and applied to C2C12 myotubes that were differentiated for five days. Compared to the myotubes grown in normal differentiation media (Figure 8a) and the conditioned media of C26 cells with control challenge (Figure 8b), the flagellin-challenged conditioned media (Figure 8c) started to deteriorate and dedifferentiate the multinucleated myotubes into mononucleated myoblasts at 48 h. The deterioration was seen via detachment of the myotubes, which may involve programmed or non-programmed cell death. However, no statistically significant differences in the myotube count between the treatments were found at this time-point. At 72 h, compared to the media of the vehicle-treated C26 cells, the conditioned media of the flagellin-treated C26 cells significantly decreased the number of myotubes by two-fold (*p* = 0.03, Figure 8d). 

### 2.7. Myeloid-Derived Suppressor Cells, M2-Type Macrophage Markers, Cancer, Cachexia, and Flagellin

**Figure 9:**
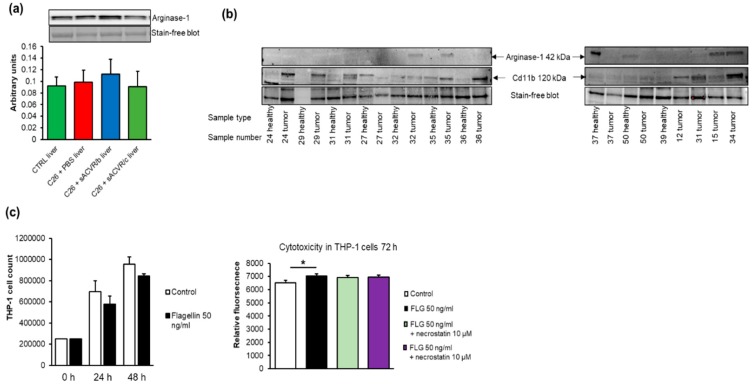
(**a**) In murine livers, no differences between the groups were found in the expression of M2-type macrophage marker Arginase-1. (**b**) Cd11b+ was found in 66% of the human tumors, with a greater prevalence in left-sided than right-sided tumors, and Arginase-1 was detected in 30% of the tumors. (**c**) Flagellin exposure started to decrease the number of THP-1 cells after 24 h, resulting in increased cytotoxicity at 72 h that was not affected by necrostatin. The blots were cut horizontally into two parts to analyze Arginase-1 and Cd11b from the same run. The original blots and the quantified intensities of the bands are provided in the Supplemental file. * Denotes a statistically significant difference between the groups that are connected with line. FLG = flagellin; control (CTRL, *n* = 9), cancer (C26 + PBS, *n* = 7), the group that received sACVR2B until tumor formation (C26 + sACVR/b, *n* = 7), and the group that received sACVR2B until death (C26 + sACVR/c, *n* = 8).

The expansion of myeloid-derived suppressor cells (MDSC) that can differentiate into M2-type macrophages has been shown to play an important role in the induction of a hepatic acute-phase protein response and an altered host energy and fat metabolism during cancer cachexia [42]. We aimed to detect the MDSC markers Cd11b+ and Gr-1 in murine liver tissue because we previously observed that they were increased in the spleen of the C26 cancer and cachexia model [26]. Unfortunately, a constitutive expression of Cd11b in hepatic Kuppfer cells impeded the assessment of MDSC in liver tissue (data not shown). The expression of the M2 macrophage marker Arginase-1 in the liver was not affected by the tumor or the treatments (Figure 9a). The Western blot analysis showed that Cd11b was found in 66% of the human tumors, being more prevalent in the left-sided than right-sided tumors, and Arginase-1 was detected in 30% of the tumors (Figure 9b).

To mimic MDSCs in vitro, we used the monocytic leukemia cell line THP-1. Flagellin exposure of THP-1 cells started to decrease the cell count at 24 h, resulting in an increased cytotoxicity after 72 h (*p* = 0.006, Figure 9c). The cytotoxicity was not mediated by RIP-kinases because the RIP-kinase inhibitor necrostatin did not affect the flagellin-induced effects (Figure 9c).

## 3. Discussion

Cachexia is a complex multi-organ syndrome occurring in ~50–80% of cancers, which is characterized by involuntary weight loss, weakness, muscle atrophy, fat depletion, anorexia, and inflammation [43]. While preclinical mouse models showed an altered gut microbiota composition in cachexia [6,44], no human studies have been conducted in this context. However, a sign of a compromised gut function in cachexia was suggested by Bindels and co-workers, who showed that, compared with non-cachectic human cancer patients, cachectic patients had higher serum levels of lipopolysaccharide binding protein, which is an indication of increased gut permeability [7]. This gut barrier dysfunction was mimicked by more direct observations in cachectic C26 tumor-bearing mice [7] and in an ApcMin/+ mouse model of colon cancer [45]. This suggests that even though the tumors are not located gastrointestinally, the C26 tumor-bearing mice had similarities to colon cancer regarding gut functions. Increased gut permeability can lead to chronic systemic inflammation [46] due to the leakage of entire bacteria or their surface molecules into the circulation. Previously, these researchers also successfully treated preclinical, cachectic cancer models with probiotic bacteria or prebiotics that modulated the gut microbiota [6,47]. However, despite these important pieces of work, it is not clear whether the gut microbiota is the cause or the consequence of cachexia. We therefore studied the gut microbiota composition of an experimental mouse C26 cancer model with an ectopically implanted tumor, with or without a blocker of activin receptor ligands, using soluble ACVR to ameliorate cachexia. While the sACVR administration improved the survival and alleviated cachexia [26], which was in agreement with results published by others [27], it had only a marginal effect on the gut microbiota composition. Our results thus suggest that ameliorating cachexia by blocking activin receptor ligands does not have a major influence on the abundance of cancer-associated microbes. Furthermore, our results suggest that the activins upregulated in C26 tumors [26] do not play a major role in the modulation of cancer-associated microbes, at least in C26 tumor-bearing mice. One of the possible causes for the changes in the gut microbiota is the increased systemic IL6 [26], as shown by Bindels et al. in C26 tumor-bearing mice [7], or perhaps some systemic metabolic changes in these mice [48].

Because many flagellated taxa were increased in the tumor-bearing mice, and due to the recent observation that bacterial flagellin is associated with CRC risk [17], we studied the direct effects of flagellin on C26 cancer cells in vitro. We first concentrated on inflammation because of the importance of the inflammatory tumor microenvironment in CRC carcinogenesis [49]. The proteins CCL2/MCP-1 and IL6 are among the most prevalent cytokines in tumor microenvironment, with increased expression generally linked with cachexia [15], tumor growth, and the survival of myeloid monocytes recruited to the tumor microenvironment and their differentiation toward tumor-promoting M2-type macrophages [50]. Both cytokines were also elevated in the sera of the present C26 mice [26]. Flagellin exposure of C26 cells increased the expression of *CCL2* and *IL6* mRNA in agreement with the previously reported effects of flagellin on osteoblastic cells [51] and basophils [52]. CCL2 was suggested to induce the production and excretion of IL6 [33], and our results reproduce these findings. While flagellin exposure markedly induced IL6 excretion, simultaneous blocking of CCL2 with a neutralizing antibody halved this response, indicating that the effects of flagellin on IL6 were partially mediated by CCL2.

In addition to IL6 and CCL2, flagellin increased the expression of *NLRP3* in C26 cells. Flagellin that reaches the cytosol is bound by the NLRC4 inflammasome, which further recruits NLRP3 [21]. The role of the NLRP3 inflammasome in cancer is controversial. On one hand, it has been shown to enhance the proliferation of the lung adenocarcinoma A549 cell line, hepatocellular carcinoma, and the formation of metastases [53]. On the other hand, it has been proposed to suppress the metastatic growth of CRC in liver [54]. Inflammasomes activate caspase-1, which can act as a tumor suppressor to regulate proliferation and apoptosis [55]. Unexpectedly, we found that despite enhancing *NLRP3,* flagellin decreased caspase-1 activity in C26 cells. This was likely due to IL6 being such a potent signal for apoptosis evasion [36] that even when NLRP3 was highly expressed, caspase-1 activity decreased.

Cancer cells that manage to evade apoptosis tend to undergo necrosis or necroptosis and autophagy, which can promote tumor growth and survival [39]. Unlike apoptosis, which is tumor-suppressive, necrosis is an unprogrammed “reparative cell death” [56] that is highly implicated in CRC pathogenesis [57]. Our results suggest that flagellin induced necrosis but not necroptosis, because it increased cytotoxicity in vitro both in C26 and THP-1 cells without affecting caspase-8 activity. Further, this was not mediated by RIP-kinases that are involved in “programmed” necroptosis via caspase-8 activity [40]. In addition, flagellin tended to increase the expression of p62, a receptor that recognizes toxic cellular waste and targets specific cargoes for autophagy [41]. The possible mechanism for the caspase-1 and inflammasome-independent cell death induced by cytosolic flagellin was suggested to be lysosomal cell death [58]. Unlike inflammasome-induced pyroptosis, this unusual form of cell death induces a mixed necrotic or sub-apoptotic semblance and retains an inflammatory outcome [58]. Another mechanism through which cancer cells can evade apoptosis is by the decrease in ROS levels by increasing antioxidant capacity [59]. Indeed, a reduced production of ROS was detected in response to flagellin exposure.

As previously mentioned, unfortunately, in vivo measurement of flagellin levels is challenging, in part because its appearance in blood and organs is sporadic and transient [17,35]. Therefore, we retrospectively determined its possible role in murine and human tumors. Mimicking the effects of flagellin on cultured CRC cells, both *IL6* and *CCL2* were significantly increased in murine C26 cancer tumors when compared with the C26 tumor cells from which the C26 tumors originated. The amelioration of cachexia by sACVR treatment did not affect the expression of these cytokines. Compared with the healthy human colon sites, the expression of CCL2 was significantly increased and IL6 tended to be increased in CRC tumors at the pathological site of the colon. However, the tumor heterogeneity in terms of primary tumor size, lymph nodes involved, and the number of metastases is a weakness of the human samples and may have affected the results. Despite the fact that the sACVR-treated mice did not consistently differ from the untreated cancer mice, it is interesting that the in vitro conditioned media from the flagellin-treated C26 cancer cells deteriorated and dedifferentiated murine myotubes, resulting finally in the reduction of viable myotubes. This indicates that flagellin might also play a role in the previously observed impaired regeneration in cancer cachexia [60]. However, the mechanisms that regulate the gut–muscle axis are largely unknown [61]. Moreover, the mechanisms of the possible minor effects of the continued blocking of activins and myostatin on the gut microbiome are not known. Because C26 tumors induce a large upregulation of activin A expression [26], sACVR might induce some effects either directly or indirectly on the gut through blocking activin A. This is because activin A has various effects on mammalian physiology and it is, together with its receptor, expressed in the gut [62]. Altogether, our results warrant further studies to determine the importance of flagellin exposure in human and mouse CRC and cachexia in vivo.

In line with the results presented by Bindels et al. [7] using the C26 cancer model, the relative abundance of the phylum Bacteroidetes was decreased in the untreated tumor-bearing mice when compared to healthy mice. However, while our results at the genus level showed an increased relative abundance of *Lactobacillus* in these mice, previous studies demonstrated that several *Lactobacillus* strains exert anti-proliferative, immunomodulatory, and antioxidant effects on cancer cells in vitro [63] and in vivo [64]. The *Lactobacillus* genus includes probiotic bacteria that, among other “good” characteristics, have the ability to aggregate with another organism in the gut. It was shown that several species of this genus can degrade biofilms formed by *Aggregatibacter* [65] and are present in biofilms together with Porphyromonadaceae [66]. Because in our study these taxa were also more abundant in the C26 cancer model, especially in the discontinued treatment group (sACVR/b) with attenuated survival [26], it may be that biofilm formation attracts *Lactobacillus*, whose abundance then increases in cancer. *Aggregatibacter* is an interesting taxon, including several oral pathogens that have been associated with pancreatic cancer [67]. *Aggregatibacter* belongs to the Pasteurellaceae family, whose abundance was elevated in the tumor-bearing mice of our study. Earlier studies in C26 mice did not report this increase, but Pasteurellaceae was shown to be increased in CRC patients [68]. There are important gaps in our understanding of how a bacterium predominantly found in the oral cavity can migrate to the colon. However, in addition to *Aggregatibacter,* another oral pathogen, *Fusobacterium nucleatum,* was associated with CRC. *F. nucleatum* was found in mutualistic biofilms together with *Aggregatibacter* [69], and it was suggested that Gal-GalNAc, a carbohydrate moiety overexpressed on CRC cells, as well as dysplastic and neoplastic lesions of the colon, facilitates *F*. *nucleatum* binding. Nevertheless, the mechanisms that enable the migration of *Aggregatibacter* to the colon are by far unknown.

We found an enrichment of the Enterococcaecae family and the *Enterococcus* genus in the C26 tumor-bearing mice in agreement with a recent study [7]. *Enterococcus* bacteremia has been suggested to be a manifestation of CRC [70]. With the presence of intestinal lesions or tumor, the gut epithelium is compromised and the gut microbes including. *Enterococcus faecalis* can translocate into the bloodstream and cause infection or even endocarditis [71]. Interestingly, as a facultative anaerobe, *E. faecalis* is also adapted to the oral cavity [72], highlighting again the importance of oral microbes in cancer. While in our experimental murine study the tumor did not originate from the colon, rather, the C26 colon tumor cells [73] were implanted subcutaneously, it is interesting that *Lachnospiraceae*, whose relative abundance was increased in the tumor-bearing mice, was reported to be depleted in C26 cancer cachexia [44] and colon adenoma patients [74]. *Lachnospiraceae* includes several taxa that are associated with good intestinal health, such as *Faecalibacterium prausnitzii* [75]. However, the relative abundance of *Lachnospiraceae* was higher in undernourished than in obese children, and was associated negatively with energy consumption [76]. Thus, it may be that in our cachectic C26 model with decreased energy intake [26], the increase in the relative abundance of *Lachnospiraceae* is in response to a lower energy intake. The physiological relevance of this finding needs further study.

## 4. Materials and Methods

### 4.1. Animals and Experimental Design

Experimental 5–6-week-old male BALB/cAnNCrl mice (Charles River Laboratories, Germany) were housed under standard conditions (temperature 22 °C, 12:12 h light/dark cycle) with free access to food pellets (R36; 4% fat, 55.7% carbohydrate, 18.5% protein, 3 kcal/g, Labfor, Stockholm Sweden) and water.

The animal treatment and care complied with the European Convention for the protection of vertebrate animals used for experimental and other scientific purposes. The protocols were approved by the National Animal Experiment Board, and all the experiments were carried out in accordance with the guidelines of that committee (permit number: ESAVI/10137/04.10.07/2014) and with the ethical standards laid down in the 1964 Declaration of Helsinki and its later amendments.

The colon 26 carcinoma (C26) cells were maintained in complete Dulbecco’s Modified Eagle’s Medium (DMEM) (high glucose, GlutaMAX™ Supplement, pyruvate, Gibco™, Life Technologies, Carlsbad, CA, USA) supplemented with penicillin (100 U/mL), streptomycin (100 μg/mL) (P/S), and 10% FBS.

As previously described [26], the mice were randomized and divided into four groups: (1) healthy control mice (CTRL), (2) C26 tumor-bearing mice receiving vehicle treatment throughout the experiment (C26 + PBS), (3) C26 tumor-bearing mice receiving sACVR treatment before tumor formation (until day one after C26 cell inoculation), followed by vehicle treatment until the end of the experiment (C26 + sACVR/b), and (4) C26 tumor-bearing mice receiving continued sACVR treatment throughout the experiment (C26 + sACVR/c). For the cancer cell inoculation, the mice were anaesthetized by ketamine and xylazine (Ketaminol^®^ and Rompun^®^, respectively) and inoculated with 5 × 10^5^ C26 cells in 100 µL of PBS (tumor-bearing mice) or with an equal volume of vehicle (PBS) only (CTRL) into the intrascapular subcutis at day 0. sACVR (5 mg/kg) or PBS (100 µL) were administered intraperitoneally twice a week, three times before and three times after C26 cell inoculation (on days −11, −7, −3, 1, 5, and 9).

### 4.2. Human Tumor Samples

The human tumor samples were derived from patients treated for CRC with surgery at the Central Finland Central Hospital. The Ethics committee of the Central Finland Health Care District (Document number 2U/2019) approved the use of the remaining tumor sample for research, following diagnostic procedures. During surgery, a specimen was also taken from a healthy site of the colon. The age and gender of the patients, as well as general characteristics of the tumors, are presented in Table 2.

### 4.3. The Production of sACVR

The recombinant fusion protein was produced and purified as described earlier in detail [77]. In short, the ectodomain of human ACVR2B was fused with a human IgG1 Fc domain and expressed in Chinese hamster ovary cells, which were grown in a suspension culture.

### 4.4. Fecal DNA Extraction, 16S rRNA Sequencing and Analysis of the Gut Microbiota Composition

At day 11 of the experiment, the mice were anaesthetized by an intraperitoneal injection of ketamine and xylazine (Ketaminol^®^ and Rompun^®^, respectively), and euthanized by cardiac puncture followed by cervical dislocation. The colon contents were collected, snap-frozen in liquid nitrogen, and stored at −80 °C. DNA was extracted with semi-automated GenoXtract and stool extraction kit (Hain Lifesciences, Nehren, Germany) accompanied with an additional homogenization by bead-beating in 1.4 mm Ceramic Bead Tubes (MO BIO Laboratories, Inc., Carlsbad, CA, USA). The microbiota composition was analyzed with next generation sequencing as previously described [78]. Briefly, the V4 region of the bacterial 16S rRNA gene was amplified using KAPA HiFi PCR kit (KAPA Biosystems, Wilmington, MA, USA) and custom-designed indexed primers (purchased from biomers.net). The PCR products were purified with Agencourt AMPure XP Magnetic beads (Beckman Coulter, Inc., Brea, CA, USA) utilizing DynaMag™-96 magnenic plate (Invitrogen, Carlsbad, CA, USA). The product length and DNA integrity were checked with TapeStation (Agilent Technologies Inc., Santa Clara, CA, USA), and the final DNA concentrations were measured with Qubit 2.0 fluorometer (Invitrogen). The sequencing was performed using the Illumina Miseq system. The quality of the sequence data was checked using the fastQC program (http://www.bioinformatics.babraham.ac.uk/projects/fastqc/), and the dataset and statistics were analyzed using the QIIME 1.9 pipeline [79]. The sequence reads were filtered using a quality score acceptance threshold 20. Chimeric sequences were filtered using usearch (v. 6.1), and OTUs were picked using the uclust algorithm with 97% sequence similarity. OTUs representing less than 0.005% of the total sequence count were excluded. Then, in order to minimize the effect of inter-sample variation in the sequencing efficiency, samples were rarefied by random sampling without replacement to the lowest common sequencing depth (22,926 reads). Annotations for the resulting OTUs were derived from the GreenGenes database [80].

### 4.5. C26 and Thp-1 Cell Cultures, Flagellin Exposure, and Receptor Blockage Using Anti-CCL2 and TH1020 Antagonist

The THP-1 monocytic cell line (*ATCC*^®^ TIB-202^™^) was a kind gift from Dr. Katariina Öörni, University of Helsinki, Finland. The cells were maintained in RPMI-1640 supplemented with 10% FBS, P/S, glutamine (all from Gibco Life Technologies), and 0.05 mm 2-mercaptoethanol (Sigma-Aldrich, St. Louis, MO, USA). To count the THP-1 cells upon flagellin exposure, we used the BioRad (Hercules, CA, USA) TC20 Automated Cell Counter. The cells from each well of the 6-well plate were counted twice.

The C26 cells were maintained as described above. When the cells were exposed to ultrapure *Salmonella typhimurium* flagellin (Invivogen, San Diego, CA, USA), FBS and P/S were retrieved from the medium. First, different doses (10 and 50 ng/mL) of flagellin and different exposure times (4 and 24 h) were tested for C26 cells, and afterward, the experiments were performed with 50 ng/mL of flagellin for 24 h. The control cells were always treated similarly with endotoxin-free H_2_O (Invivogen) into which flagellin stock solution (100 ng/µL) was prepared. To block TLR5, the specific antagonist TH1020 (Tocris, Bristol, UK) in a concentration of 0.85 or 2 µM was added to the C26 cultures 30 min prior to flagellin exposure. To neutralize the effects of CCL2, mouse polyclonal CCL2/JE/MCP-1 antibody (R&D Systems, Minneapolis, MN, USA) in a concentration of 10 µg/mL was added to the C26 cultures 30 min prior to flagellin exposure. Each treatment was repeated four times and the results were confirmed using two technical replicates (*n* = 4 × 2).

### 4.6. Measurement of IL6 Excretion, Caspase-1 and -8 Activity, Reactive Oxygen Species Production, and Cytotoxicity

To measure IL6 excretion, 1 × 10^4^ C26 cells were plated on the 96-well plates and exposed to endotoxin-free H_2_O, 50 ng/mL of flagellin, 10 µg/mL of anti-CCL2 or flagellin + anti-CCL2 the next day for 24 h. Afterward, the cell culture media was collected and centrifuged at 1200 rpm for 3 min. The IL6 was measured using the mouse IL6 ELISA kit (Invitrogen) according to the manufacturer’s instructions. For reactive oxygen species (ROS) and caspase-1 activity measurement, the C26 cells were plated as described above and exposed to endotoxin-free H_2_O or 50 ng/mL of flagellin for 1, 4, and 24 h (for ROS) or for 1 h (for caspase-1).

ROS production, caspase-1, and caspase-8 activity were measured according to the manufacturer’s instructions using the Promega GloMax system (Promega, Madison, WI, USA), with the ROS-Glo H_2_O_2_ Assay, the Caspase-Glo-1, and the Caspase-8 Inflammasome Assay (Promega), respectively. The ROS-Glo™ H_2_O_2_ Assay is a bioluminescent assay that measures the level of hydrogen peroxide (H_2_O_2_), a reactive oxygen species (ROS). A derivatized luciferin substrate is incubated with the sample and reacts directly with H_2_O_2_ to generate a luciferin precursor. The produced light signal is proportional to the level of H_2_O_2_ present in the sample. In the Caspase-Glo Assay, the selective caspase substrate enables the detection of catalytically active caspase in cells and quantitatively measures inflammasome activity. The assay includes a caspase-specific inhibitor to confirm the specific activity and distinguish caspase-1 or caspase-8 activity from other caspases.

For cytotoxicity measurements, C26 cells were plated as described above and exposed to endotoxin-free H_2_O or 6.25, 12.5, 25, and 50 ng/mL of flagellin for 24, 48, and 72 h. To measure cytotoxicity in THP-1 cells, the cells were exposed to 50 ng/mL of flagellin for 72 h with or without RIP-kinase inhibitor necrostatin (10 or 60 µM, Sigma Aldrich). Cytotoxicity was measured using the endpoint method of CellTox Green Cytotoxicity Assay and Promega GloMax system (Promega), as specified by the manufacturer. This assay measures changes in membrane integrity that occur because of cell death. To study whether the cytotoxicity in C26 cells was mediated by RIP-kinases, a specific kinase inhibitor, necrostatin, was added together with flagellin in a concentration of 10 or 60 µM.

### 4.7. C2C12 Myotubes, Treatment with Conditioned Media from C26 Cells, and Analysis of Myotube Sizes and Numbers

C2C12 myoblasts (ATCC, Manassas, VA, USA) were plated on the 12-well plates with collagen-coated coverslips in DMEM + GlutaMax (high glucose + pyruvate) supplemented with 10% FBS and penicillin/streptomycin (P/S) (all from Invitrogen). After two days, when the myoblasts had reached ~90% confluence, a differentiation media replaced the cells. In the differentiation media, FBS was replaced by 2% horse serum (Invitrogen). C26 cells were treated with 50 ng/mL of flagellin or vehicle for 24 h. Afterward, the media was collected, centrifuged for 3 min at 1500 rpm, and supplemented with 2% of horse serum. At day five of the C2C12 myotube differentiation, the conditioned C26 cell media was applied to the C2C12 myotube cultures for 48 and 72 h. The myotubes were fixed with 4% paraformaldehyde/PBS for 5 min at RT, then washed with PBS. Standard hematoxylin/eosin (H&E) staining was used to stain and visualize the myotubes. The number of myotubes in each coverslip was counted manually from three to five (depending on the extent of detachment of myotubes) random fields that, with 4 X magnification, practically covered the entire coverslip. The scoring of the myotubes vs. the dedifferentiated myoblasts was based on whether they were multinucleated or mononucleated, respectively. 

### 4.8. RNA Extraction, Quantitative Real-Time PCR, Protein Extraction from Cells and Human Tumors, and Western Blot

The total RNA from the cells and tumors was extracted using Tri reagent (Thermo Fisher, Waltham, MA, USA) according to the supplier’s protocol. Real-time PCR analysis was done according to MIQE guidelines using in-house designed primers (from Invitrogen), iq SYBR Supermix, and CFX96™ Real-time PCR Detection System (Bio-Rad Laboratories, Hercules, CA, USA). *Actb* was used as a housekeeping gene to normalize the results. No differences in the mRNA levels of *Actb* were detected between the groups. The primer sequences were as follows: *NLRP3* fwd 3′AAAATGCCTTGGGAGACTCA5′, *NLRP3* rev 3′AAGTAAGGCCGGAATTCACC5′, *IL6* fwd 3′ CTGATGCTGGTGACAACCAC5′, *IL6* rev 3′CAGAATTGCCACATTGCACAAC5′, *CCL2* fwd 3′ AGGTGTCCCAAAGAAGCTGTA5′, *CCL2* rev 3′ATGTCTGGACCCATTCCTTCT5′, *Actb* fwd 3′GGCTGTATTCCCCTCCATCG5′, *Actb* rev 3′CCAGTTGGTAACAATGCCATGT5′.

The total proteins from the cells and tumors were extracted using ice-cold lysis buffer (20 mm Hepes, pH 7.4, 1 mm EDTA, 5 mm EGTA, 10 mm MgCl_2_, 100 mm β-glycerophosphate, 1 mm Na_3_VO_4_, 1 mm DTT, 1% Triton-X100, supplemented with protease and phosphate inhibitors (Sigma Aldrich)). The hepatic protein extracts were previously prepared [26]. Western blots were done by purchasing the primary antibodies from Cell Signaling Technology (Danvers, MA, USA) and R&D Systems, and by scanning the blots using the ChemiDoc MP Imaging System (BioRad). Odyssey anti-rabbit IRDye 800CW and anti-mouse IRDye 680RD (LI-COR Biosciences, Lincoln, NE, USA) were used as secondary antibodies. The quantified bands were normalized to the total protein content per well, as visualized by using stain-free precast Criterion 4–20% gradient gels (BioRad).

### 4.9. Statistical Analysis

Normal distribution of the data was assessed using the Shapiro Wilk’s test in IBM SPSS Statistics version 24 for Windows (SPSS, Chicago, IL, USA). The analyses of the normally distributed data were conducted using the general linear model analysis of variance (ANOVA), followed by the Bonferroni post hoc test. The analyses of the non-normally distributed data were conducted using the non-parametric Mann–Whitney U test. The level of significance in all analyses was set at *p* < 0.05. The statistical analyses were performed with IBM SPSS Statistics version 24 for Windows, except for the gut microbiota composition differences between the groups, which were analyzed using the Kruskall–Wallis test in the QIIME 1.9 pipeline [79]. The statistical significance of the gut microbiota findings was corrected for the Benjamini–Hochberg False Discovery Rate (FDR). The data were expressed as means ± SD, if not otherwise mentioned. Spearman’s correlation coefficients followed by FDR correction were used to analyze correlations between the microbiota and body weight loss. The heat map analysis of the correlations was conducted using Clustvis, a web tool for visualizing clustering of multivariate data (https://biit.cs.ut.ee/clustvis/). In the heat map, the columns were clustered using correlation distance and average linkage. The groups were analyzed separately and together. In the heat map, only the separate analysis was shown. The associations between the other cachectic features and flagellated microbes were analyzed using Spearman’s correlation coefficients. Here, FDR correction was not used, because only a few selected microbial taxa were included in the analysis.

## 5. Conclusions

Our study showed that the abundance of flagellated microbes was increased by the C26 experimental cancer in mice. Flagellated microbes may play a role in cachexia and shaping the inflammatory micro-environment of colorectal cancer. The in vitro results suggested that flagellin promotes the survival of colon cancer cells by inhibiting apoptosis and by inducing inflammatory cytokines, such as CCL2/MCP-1, as well as necrosis. A similar cytokine induction was also detected in murine and human tumors. The conditioned media from the flagellin-treated cancer cells deteriorated the myotubes and decreased their number, suggesting that flagellin may also contribute to impaired regeneration in cachexia. The amelioration of cachexia in mice by blocking activin receptor ligands did not have a major influence on the overall composition of the microbiota. However, the continued treatment of cachexia modified the associations between the gut microbiota and body weight loss to more resemble those of healthy mice, warranting a need for further study regarding blocking activin receptor ligands and the gut microbiota. In addition, deeper studies are needed in the future from cancer patients, in which methods to quantify flagellar genes from feces and to link the genes to certain bacterial species should be used.

## Figures and Tables

**Table 1:** The statistically significant differences and the direction of the change in the taxa between the C26 + PBS and CTRL mice.

Taxon	C26 + PBS vs. CTRL *p*-Value	C26 + PBS vs. CTRL Direction
**Phylum**		
Bacteroidetes	0.046	↓
Deferribacteres	0.046	↑
Firmicutes	0.046	↑
Proteobacteria	0.046	↑
**Family**		
Bacteroidaceae	0.028	↑
Deferribacteraceae	0.034	↑
Lactobacillaceae	0.005	↑
Pasteurellaceae	0.005	↑
Porphyromonadaceae	0.005	↑
Enterococcaceae	0.007	↑
Lachnospiraceae	0.009	↑
S24-7	0.023	↓
Prevotellaceae	0.007	↓
**Genus**		
*Bacteroides*	0.030	↑
*Lactobacillus*	0.008	↑
*Enterococcus*	0.011	↑
*Mucispirillum*	0.037	↑
ud*-Lachnospiraceae*	0.012	↑
ud*-S24-7*	0.024	↓
*Prevotella*	0.011	↓
*Aggregatibacter*	0.008	↑

Abbreviations and signs: ud denotes unidentified, ↑ means increased compared to CTRL, and means ↓ decreased.

**Table 2:** The characteristics of the human patients and tumors used in the study. pT and pN according to TNM Classification of Malignant Tumors (TNM); p is the stage given by histopathological examination, T is the size of the primary tumor, and N the nearby lymph nodes involved.

Sample	Tumor Location	Gender	Age	Diagnosis	pT	pN	Metastases
12	Sigma	Female	58	Adenocarcinoma	a	pN1b	3/10
15	Rectum	Female	65	Adenocarcinoma	pT4	pN0	0/6
24	Sigma	Male	78	Adenocarcinoma	pT4a	pN2b	15/35
27	Caecum	Male	84	Adenocarcinoma	pT4b	pN2a	4/15
29	Colon (ascendens)	Male	91	Adenoma tubulovillosum			
31	Colon (ascendens)	Female	69	Adenoma tubulovillosum			
32	Caecum	Female	59	Adenocarcinoma	pT3	pN2b	8/16
34	Caecum	Male	79	Adenoma tubulare			
35	Caecum	Male	56	Adenocarcinoma	pT2	pN0	0/22
36	Sigma	Female	68	Adenocarcinoma	pT4a	pN0	0/13
37	Sigma	Male	67	Adenocarcinoma	pT2	pN0	0/13
39	Sigma	Female	70	Adenocarcinoma	pT3	pN0	0/13
50	Sigma	Male	65	Adenocarcinoma	pT3	pN2b	11/38

## Supplementary Materials

The following are available online at https://www.mdpi.com/2072-6694/11/11/1799/s1, The original blots and the quantified intensities of the bands.
