# Supplementary material for: Blocking Activin Receptor Ligands Is Not Sufficient to Rescue Cancer-Associated Gut Microbiota—A Role for Gut Microbial Flagellin in Colorectal Cancer and Cachexia?
Source: Cancers (Basel). 2019 Nov 15;11(11):1799. doi: 10.3390/cancers11111799 (PMC6896205; doi:10.3390/cancers11111799)
Supplement: Supplementary file 1 [file cancers-11-01799-s001.pdf]

Supplementary Files:

Blocking Activin Receptor Ligands Is Not Sufficient to Rescue Cancer-Associated Gut Microbiota—A Role for Gut Microbial Flagellin in Colorectal Cancer and Cachexia?

Satu Pekkala, Anniina Keskitalo, Emilia Kettunen, Sanna Lensu, Noora Nykänen, Teijo Kuopio, Olli Ritvos, Jaakko Hentilä, Tuuli A. Nissinen and Juha J Hulmi

S1

Arginase 1 (42 kDa) mouse liver, secondary antibody IRDye800CW

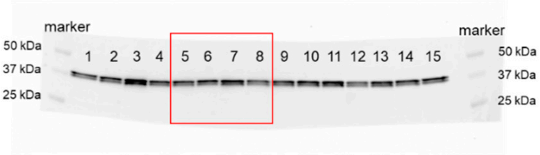

Arginase 1 mouse liver, quantification

Lane No	Sample ID	Group	Band intensity volume
1	12	CTRL	7280531
2	14	C26 + PBS	7701750
3	27	C26 + sACVR/b	11598519
4	22	C26 + sACVR/c	6788350
5	23	CTRL	6292915
6	15	C26 + PBS	6481558
7	28	C26 + sACVR/b	9212761
8	31	C26 + sACVR/c	5693269
9	24	CTRL	7342761
10	16	C26 + PBS	8398853
11	30	C26 + sACVR/b	9483623
12	35	C26 + sACVR/c	5247871
13	33	CTRL	7618565
14	36	C26 + sACVR/c	7404183
15	34	CTRL	8550099

Stain free blot, mouse liver

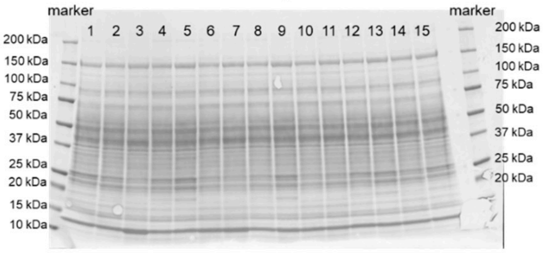

Stain free blot, quantification

Lane No	Sample ID	Group	Lane intensity volume
1	12	CTRL	147845269
2	14	C26 + PBS	155830194
3	27	C26 + sACVR/b	155607375
4	22	C26 + sACVR/c	171221540
5	23	CTRL	158662580
6	15	C26 + PBS	155010600
7	28	C26 + sACVR/b	148735905
8	31	C26 + sACVR/c	144642967
9	24	CTRL	157757040
10	16	C26 + PBS	133762809
11	30	C26 + sACVR/b	143218278
12	35	C26 + sACVR/c	163273867
13	33	CTRL	175862862
14	36	C26 + sACVR/c	191830496
15	34	CTRL	166270050

S1. The original blots that have been used to make Figure 9a, and their quantification with ChemiDoc MP Imaging System and software. As a marker, BioRad All Blue was used. The lanes used for Figure 9a are marked with surrounding red line.

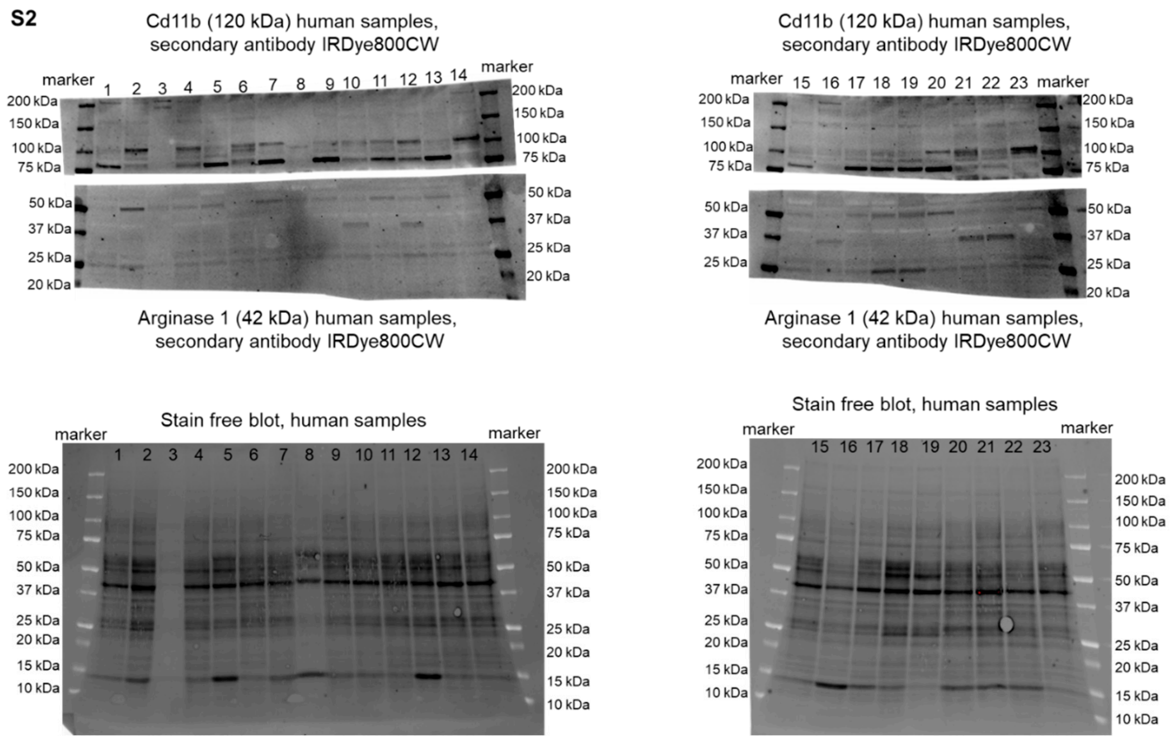

Cd11b, human samples, quantification

Lane No	Sample ID	Sample type	Band intensity volume
1	24	healthy site	45069
2	24	tumor	244989
3	29	healthy site	294
4	29	tumor	122255
5	31	healthy site	49073
6	31	tumor	108272
7	27	healthy site	110162
8	27	tumor	244
9	32	healthy site	49684
10	32	tumor	41140
11	35	healthy site	57763
12	35	tumor	114560
13	36	healthy site	21183
14	36	tumor	204061
15	37	healthy site	28756
16	37	tumor	28049
17	50	healthy site	27227
18	50	tumor	50843
19	39	healthy site	11672
20	12	tumor	129915
21	31	tumor	178496
22	15	tumor	93052
23	34	tumor	414324

Arginase 1, human samples, quantification

Lane No	Sample ID	Sample type	Band intensity volume
1	24	healthy site	-71694
2	24	tumor	182670
3	29	healthy site	-20916
4	29	tumor	-25417
5	31	healthy site	70378
6	31	tumor	-121330
7	27	healthy site	-152569
8	27	tumor	104871
9	32	healthy site	-66268
10	32	tumor	1327754
11	35	healthy site	-60086
12	35	tumor	1533488
13	36	healthy site	-7560
14	36	tumor	-119406
15	37	healthy site	11686
16	37	tumor	1506804
17	50	healthy site	95398
18	50	tumor	178748
19	39	healthy site	-48922
20	12	tumor	350739
21	31	tumor	3314165
22	15	tumor	3373603
23	34	tumor	430636

Stain free blot, human samples, quantification

Lane No	Sample ID	Sample type	Lane intensity volume
1	24	healthy site	220652218
2	24	tumor	431873737
3	29	healthy site	45512989
4	29	tumor	392854175
5	31	healthy site	341286857
6	31	tumor	354136484
7	27	healthy site	277783350
8	27	tumor	69677368
9	32	healthy site	263447236
10	32	tumor	337963028
11	35	healthy site	339313664
12	35	tumor	391597573
13	36	healthy site	351478168
14	36	tumor	430181081
15	37	healthy site	228048537
16	37	tumor	310930046
17	50	healthy site	293118055
18	50	tumor	504953191
19	39	healthy site	312390913
20	12	tumor	332518607
21	31	tumor	512916363
22	15	tumor	299649765
23	34	tumor	186326291

S2. The original blots that have been used to make Figure 9b, and their quantification with ChemiDoc MP Imaging System and software. As a marker, BioRad All Blue was used.

© 2019 by the authors. Licensee MDPI, Basel, Switzerland. This article is an open access article distributed under the terms and conditions of the Creative Commons Attribution (CC BY) license (http://creativecommons.org/licenses/by/4.0/).
